# Zeolite Nanocrystals Protect the Performance of Organic Additives and Adsorb Acid Compounds during Lubricants Oxidation

**DOI:** 10.3390/ma12172830

**Published:** 2019-09-03

**Authors:** Moussa Zaarour, Hussein El Siblani, Nicolas Arnault, Philippe Boullay, Svetlana Mintova

**Affiliations:** 1 Laboratoire Catalyse et Spectrochimie, Normandie Univ, ENSICAEN, UNICAEN, CNRS, 14050 Caen, France; 2 Sogefi Group, Parc Ariane IV, 7 Avenue du 8 mai 1945, 78286 Guyancourt CEDEX, France; 3 Normandie Univ, ENSICAEN, UNICAEN, CNRS, CRISMAT, 14000 Caen, France

**Keywords:** zeolite, lubricants, additive protection, anti-oxidant, FTIR, NMR

## Abstract

Zeolite nanocrystals were used as proactive agents to extend the lifetime of commercial lubricants by protecting the performance additives from depletion and adsorbing the acid formed during oxidation. The nanosized zeolites were introduced into four lubricants and subjected to oxidation (90 °C and 150 °C). A strong affinity towards protection of zinc dialkyldithiophosphate (ZDDP) additive was demonstrated by ^31^P NMR (nuclear magnetic resonance) and FTIR (fourier-transform infrared) spectroscopy even after heating at 150 °C for 24 h. FTIR profiles of lubricants aged in the presence of LTL (Linde Type L zeolite) showed lower oxidation degree while the formed oxidation products (aldehydes, ketones, and acids) were adsorbed on the zeolite crystals acting as scavengers.

## 1. Introduction

Lubricants are indispensable in maintaining the good functioning of vehicle engines. In addition to reducing friction and wear within the metallic parts, lubricants carry out a range of other complimentary tasks including: corrosion prevention, providing a liquid seal at moving contacts, and removal of wear and soot particles [1]. On the other hand, the environmental impact resulting from their production, use, recycling, and disposal is high.

Their replacement by the more environmentally friendly vegetable oils could have been considered as an option despite of their low thermal stability and easy oxidation at elevated temperatures [1,2]. Another alternative solution is the use of synthetic lubricants that ensure a higher thermal stability, longer lifetime, and modifiable chemical and physical properties to meet the requirements. Commercial lubricants are available with special formulations; the base oil which constitutes more than 90% of the mixture is mainly responsible for lubricating the metallic parts. Its preservation, maintaining its function, and introducing other complimentary tasks is governed by the performance additives [1,3]. These are phenols, aromatic amines, alky or aromatic sulfides as antioxidants to delay the auto oxidation of the base oil [1,4], zinc dialkyldithiophosphate (ZDDP) as antiwear [1,5], poly(acrylates), olefin copolymers or styrene–butadiene copolymers as viscosity index improvers [1,6], amphiphilic molecules as detergents and dispersants to adsorb wear and soot particles and keep them in colloidal suspension in the base fluid [1,4]. While the performance additives especially the antioxidants are in function, the lubricant is said to be in the “pro-active” domain. The critical depletion of these products renders the base oil unprotected in presence of air and heat, this is the so-called “responsive domain” when it is highly advised to replace the lubricant.

In order to reduce the high environmental impact resulting from lubricant disposal, several re-refining techniques were developed including acid/clay treatment, solvent extraction, distillation/clay treatment and distillation/hydrotreatment, all of which generate chemical wastes that needs additional treatment [1]. Recycling of used lubricant can be also considered an option where unoxidized base oil is separated and recovered from the oxidized oil fraction and the depleted additives [1,7].

Despite of these end-of-use treatments, different solutions were proposed to extend the lubricant lifetime. Additives such as TiO_2_ [8,9] and Cu [10] nanoparticles, fullerenes [11], nano-diamond [12] and MoS_2_ [13] demonstrated excellent antiwear properties. Additionally, chemical filters based on mesoporous materials [14,15] or strong bases (metal oxides) [16,17,18] were developed to adsorb and neutralize acids generated from the lubricants circulating in the engine, respectively. While the incorporation of these new additives counts for extending the proactive domain, their complicated synthetic protocols and expensive starting materials create a potential limitation [3]. On the other hand, the main action of the modified chemical filters starts when the oil is already oxidizing.

Zeolites, a special class of aluminosilicate inorganic materials are known for their excellent sorption properties owing to their porous structure, high surface area, and large internal voids. The selective behavior towards gases and liquids can be tuned following a systematic modification of their composition and structure. The acidic properties of these materials are highly influenced by their form [19]. For instance, while the proton (H) form demonstrate strong Lewis and Brønsted character, the cationic zeolites present a basic character suitable to neutralize weak acids [20,21]. LTL and FAU (faujasite) nanosized zeolites have already demonstrated high capabilities as antioxidants for the preservation of palm oil during the early stages of oxidation [22,23,24]. The primary oxidation products “peroxides” are stabilized by the zeolite charge compensating cations with a strong influence driven by the type of cation used. On the other hand, the secondary oxidation products “aldehydes, ketones, and carboxylic acids” are adsorbed and immobilized on the external surface and in the internal voids of the zeolite. LTL nanocrystals were also reported for the purification of lubricants at the late stages of oxidation; the secondary oxidation products are adsorbed by the zeolite and stabilized by the strong interactions of their carbonyl groups with the potassium cations [25], consequently, they are filtered out from the non-oxidized fraction allowing its proper reuse [26]. LTL nanocrystals were further tested as anti-oxidant for synthetic lubricants in the presence or in the absence of performance additives [2]. The study revealed a strong capacity of these nanocrystals in adsorbing the oxidation products and thus delaying the formation of undesired polymers. The experiments were made on lubricants with special formulations adapted to the study, thus does not predict the exact behavior of zeolite in a commercial lubricant where numerous additives are available.

Herein we report the synthesis of LTL zeolite nanocrystals (15–20 nm) prepared from inorganic starting materials in absence of organic structural directing agents as proactive agents to extend the lifetime of commercial lubricants used for car engines. LTL nanocrystals demonstrate a dual role by (i) delaying the depletion of additives and hence extending the proactive domain, and (ii) absorbing the generated secondary oxidation products formed, and hence delays the lubricant degradation.

## 2. Materials and Methods

### 2.1. Materials

Two grades of commercial lubricants (C2 and C3) from two different suppliers (SA and SB) were provided from Sogefi group, Al(OH)_3_ was purchased from Acros Organics, Ludox SM-30 and KOH were purchased from Sigma Aldrich, dd H_2_O: double distilled water.

### 2.2. Preparation of LTL Nanocrystals

Nanosized LTL zeolite was prepared starting from the following molecular composition of the precursor suspension: 5 K_2_O, 10 SiO_2_, 0.6 Al_2_O_3_, 200 H_2_O.

In a polypropylene bottle, KOH (7.77 g) was dissolved in dd H_2_O (15 g) followed by the slow addition of Al(OH)_3_ (1.3 g). The resulting suspension was stirred overnight to afford a light white suspension which was then added in a dropwise manner over a solution of Ludox SM-30 (27.7 g) in dd H_2_O (13.7 g) under stirring. The resulting suspension was kept on a shaker for 48 h then placed in an autoclave at 170 °C for 8 h.

The crystalline material was separated from the liquid phase by centrifugation (20,000 rpm, 40 min) then purified by five cycles and redispersed in water followed by centrifugation. The LTL nanocrystals were freeze dried, then activated at 150 °C for 24 h prior to use.

### 2.3. Oxidation Process

Commercial Lubricants (100 g) were mixed with 1 wt% of LTL zeolite nanocrystals (1 g) and allowed to oxidize under stirring at 90 °C for 35 days or at 150 °C for 24 h. 5 g of each sample were withdrawn periodically; the zeolite nanocrystals were separated from the lubricants by centrifugation (20,000 rpm, 1 h). For comparison, similar amount of lubricants (100 g) was oxidized in absence of zeolite nanocrystals.

### 2.4. Characterization

#### 2.4.1. Characterization of Zeolite Nanocrystals

X-ray diffraction (XRD) characterization: the purity and crystallinity of the zeolite powder before and after lubricant oxidation were studied by XRD analyses carried out with PANalytical X’Pert Pro diffractometer with CuKα monochromatized radiation (λ = 1.5418 Å, Almelo, The Netherlands).

High-Resolution Transmission Electron Microscopy (HRTEM): the crystal size, morphology, and crystallinity were characterized using a FEI Tecnai G2 30 microscope (Vacc = 300 kV, LaB6, Eindhoven, The Netherlands).

Dynamic light scattering (DLS): the size of the zeolite nanoparticles was measured by a Malvern Zetasizer Nano instrument using a backscattering geometry (scattering angle of 173°, He-Ne laser with a 3 mW output power at a wavelength of 632.8 nm, Malvern Panalytical, Royston, UK. Analyses were performed on water suspensions with a solid concentration of 2 wt%.

Nitrogen adsorption analysis: the porosity was measured using a Micrometrics ASAP 2020 volumetric adsorption analyser (USA). Samples were degassed at 250 °C under vacuum overnight prior to the measurement. The external surface area and micropore volume were estimated by alpha-plot method using Silica-1000 (22.1 m^2^∙g^−1^ assumed) as a reference. The micropore and mesopore size distributions of samples were estimated by the Nonlocal Density Functional Theory (NLDFT) and Barret-Joyner-Halenda (BJH), respectively using the desorption branch of the isotherm.

FTIR: The zeolite nanocrystals separated from lubricants were thoroughly washed by diethyl ether to eliminate traces of physically adsorbed products and dried overnight under vacuum. The zeolite powder was diluted in KBr (1%), pressed (~10^7^ Pa) in disks (2 cm^2^ area, 100 mg∙cm^−2^), and placed in an IR cell equipped with KBr windows. IR spectra were recorded using a Nicolet 6700 IR spectrometer (Thermo Scientifics) equipped with a mercury cadmium telluride (MCT) detector and an extended KBr beam splitter. Spectra were recorded in the 400–5500 cm^−1^ range at 4 cm^−1^ with 128 scans.

#### 2.4.2. Characterization of Lubricants

^1^H NMR analyses were carried out using a Bruker AVIII spectrometer 600 MHz (Bruker, Wissembourg, France). Lubricant samples (400 mg) dissolved in deuterated chloroform (CDCl_3_) (0.5 mL) were used, and 1000 scans were applied. The chemical shifts are calibrated to residual proton resonance of Si(CH_3_)_4_ (δH = 0 ppm).

^31^P NMR analyses were carried out using a Bruker AVIII spectrometer 600 MHz. Lubricant samples (400 mg) dissolved in CDCl_3_ (0.5 mL) were used and 6000 scans were applied. The chemical shifts are calibrated to the resonance of H_3_PO_4_ (δP = 0 ppm).

Rheology: the evolution of lubricant viscosity throughout the oxidation process was monitored by a Malvern Kinexus Rheometer. The analyses were performed by applying a shear stress of 1 Pa at 25 °C. Three independent measurements were performed for each sample and the average value was used.

FTIR: The stability of the lubricants and the formation of oxidation products were monitored by FTIR spectroscopy in liquid phase using Perkin Elmer System 2000 spectrometer (Waltham, MA, USA). Lubricants (0.5 mL) were introduced in a ZnSe liquid cell (1 mm spacer) and analyzed in the 400–5500 cm^−1^ range with 128 scans.

## 3. Results and Discussion

**Figure 1:**
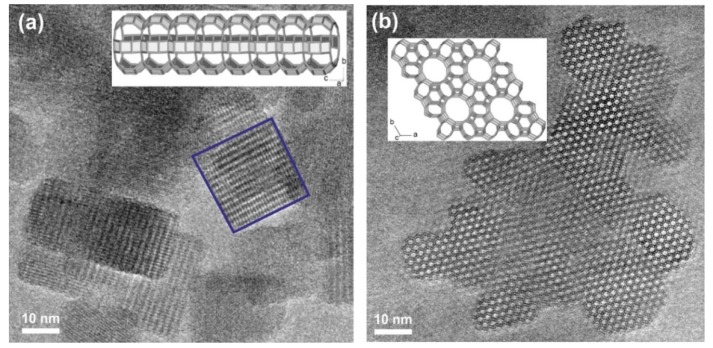
High-Resolution Transmission Electron Microscopy (HRTEM) images of LTL nanocrystals projected along the directions [100] in (**a**) and [001] in (**b**) (SG P6/mmm).

The LTL nanozeolite used in this study is prepared free of organic structural directing agents, thus avoiding energy expenditure and CO_2_ release resulting from high temperature calcinations. This zeolite was selected due to the high efficiency towards adsorbing products of lubricant oxidation [22,23,24,26], absence of Brønsted or Lewis acidity [26], hence cannot catalyze the oxidation of lubricants, in addition to the absence of toxicity on the living cells [27]. XRD pattern of the prepared zeolite reveals broad Bragg peaks typical for small nanoparticles with shifts corresponding to pure LTL structure in absence of other phases (Figure S1). The high crystallinity was further demonstrated by HRTEM that revealed particles with parallel cylindrical morphology of 15–20 nm corresponding to the 1-D structure of LTL (Figure 1). N_2_ sorption analyses (Figure S3) monitored a high surface area (454 m^2^.g^−1^) and large pore volume (0.66 cm^3^·g^−1^).

**Figure 2:**
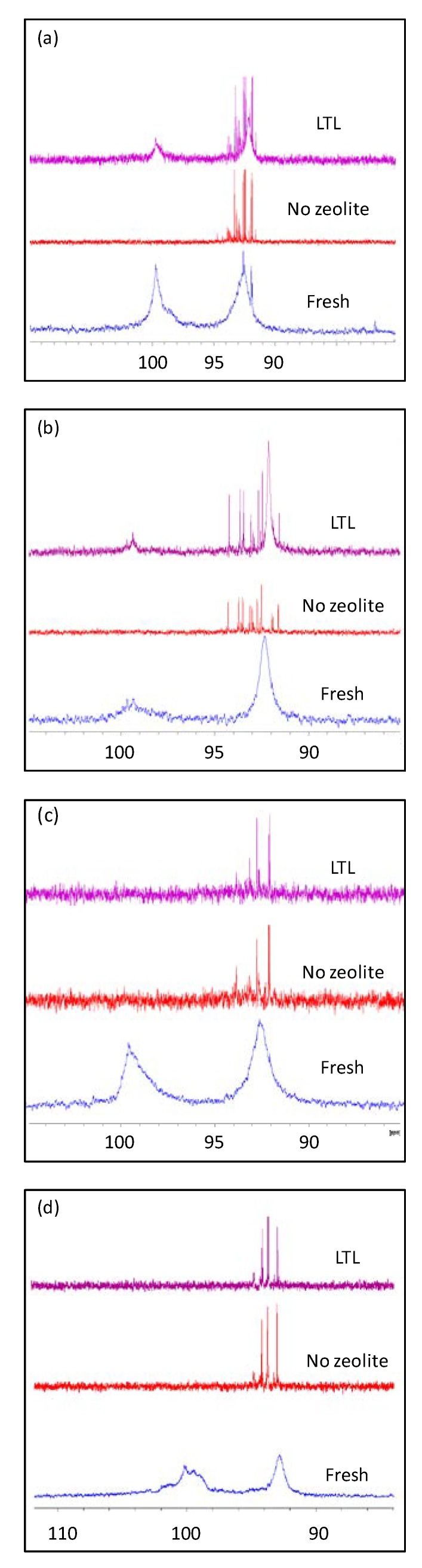
^31^P NMR spectra of (**a**) SA-C2, (**b**) SA-C3, (**c**) SB-C2, and (**d**) SB-C3 in their fresh form (blue) or following 24 h of oxidation at 150 °C in absence (red) or presence (violet) of LTL nanozeolite.

**Figure 3:**
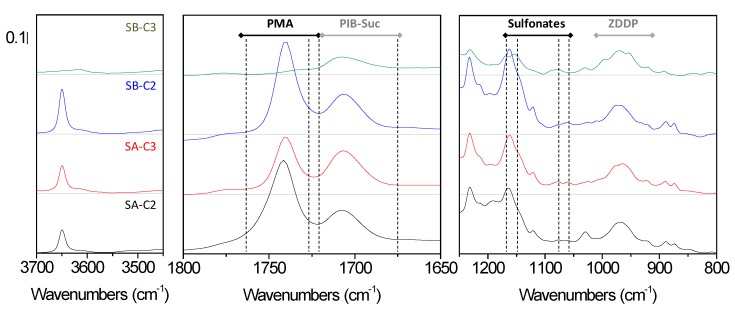
FTIR main features of fresh lubricants: SA-C2 (black), SA-C3 (red), SB-C2 (blue) and SB-C3 (green).

Four fully synthetic, commercial light lubricants (η = 0.103–0.116 Pa·s, Table S1) designed by two different suppliers for the use in car engines were tested. ^1^H NMR revealed saturated hydrocarbons constituting more than 90% of these lubricants, thus confirming their designation as fully synthetic (Figure S4). A minority of peaks was recorded in the aromatic region corresponding to alkylated phenol antioxidant in SA-C2, SA-C3, and SB-C2 in addition to an unidentified aromatic additive available in all the samples (Figure S4). The presence of phosphorous containing additives was verified by ^31^P NMR; basic and neutral ZDDP (zinc dialkyldithiophosphates) acting as antiwear and antioxidant were identified in all the samples [5] (Figure 2). The presence of numerous performance additives with different functional groups resulted in a complicated FTIR (Figure 3 and Figure S5). The following interesting features (bands) in the spectra were identified: at 975 cm^−1^ (P–O–C vibrations from ZDDP [28]), at 1705 cm^−1^ (C=O vibrartions from polyisobutylene succinimide “PIB-suc” dispersant [29]), at 1740 cm^−1^ (C=O from esters and polymethacrylates “PMA” viscosity modifiers) and at 3650 cm^−1^ (phenolic OH).

Oxidation was first conducted at 90 °C for 35 days in the absence and in the presence of 1 wt% of zeolite. All the lubricants showed high stability under these conditions, that is, no remarkable changes were identified on their viscosity, NMR or FTIR spectroscopic profiles.

**Figure 4:**
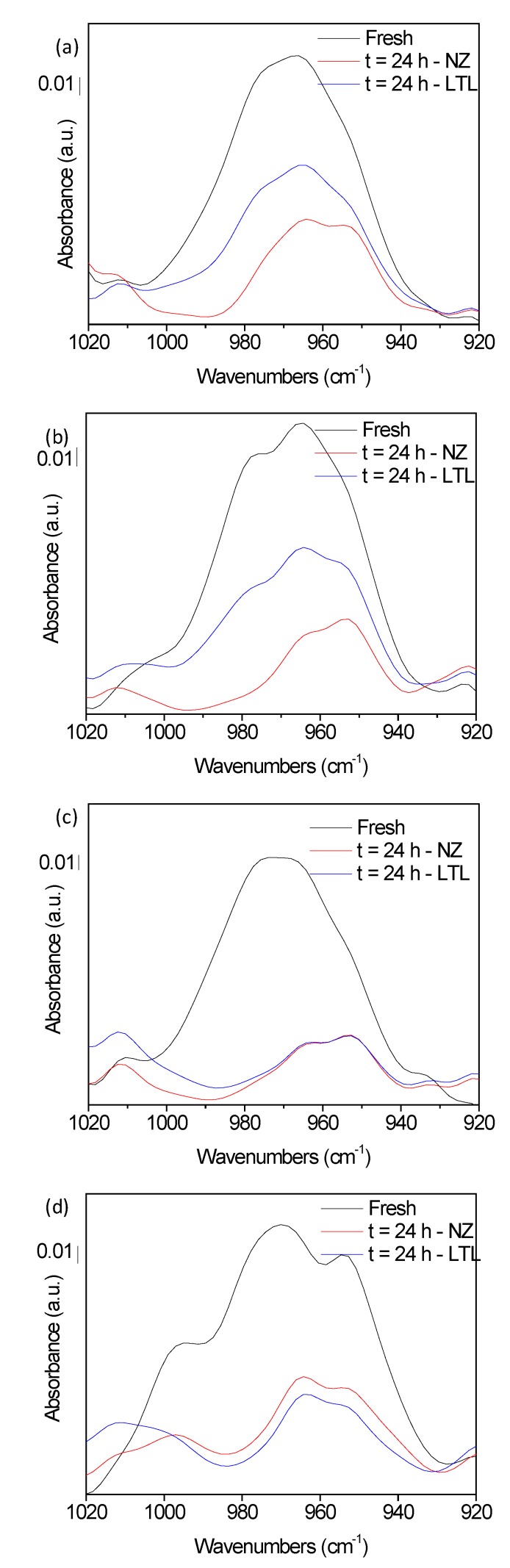
P–O–C FTIR band vibrations of (**a**) SA-C2, (**b**) SA-C3, (**c**) SB-C2, and (**d**) SB-C3 in their fresh form (black) or following 24 h of oxidation at 150 °C in absence (red) or presence (blue) of LTL nanozeolite.

A faster aging was provoked by increasing the temperature up to 150 °C. Consequently, the P–O–C FTIR vibrations of the ZDDP additive were strongly influenced evidenced by a sharp decrease of the band area from 54% to 44% for the initial sample SA-C2 and SAC3 after 16 h of heating in absence of zeolite (Figure 4 and Figure S6). Meanwhile, a further decrease to 27% and 16% for samples SA-C2 and SAC3, respectively after 24 h of oxidation was recorded. The fast depletion of the ZDDP additive was diminished in the presence of LTL nanocrystals, more precisely, 14% and 25% preservation of the P–O–C vibrations were recorded after 16 h and 24 h of heating for both samples. Notably, the efficiency of zeolite nanocrystals in limiting the loss of the FTIR vibrations increases with time; this is exemplified by the progressive increment of the difference in the P–O–C band area available after oxidation of lubricants in presence and absence of LTL as a function of time. On the other hand, both samples SB-SC2 and SB-C3 encountered a fast oxidation leading to a major loss of P–O–C vibrations after only 16 h with no remarkable influence identified for the zeolite.

The evolution of ZDDP was further examined by ^31^P NMR spectroscopy. The transformation of the broad features resulting from the tetrahedron structure of Zn metal center [30] into sharp narrow peaks at lower chemical shifts gives a clear indication of thermal degradation and oxidation [31]. Upon aging at 150 °C, the broad feature vanished completely in absence of zeolite nanocrystals (Figure 2), leading to say that the ZDDP is completely decomposed. The set of peaks present at 90–95 ppm corresponds to (RO)_2_P(S)SR’, a rearrangement product resulting from a high thermal stress of ZDDP. On the other hand, despite of the partial dissociation of ZDDP in SA-C2 and SA-C3 aged in the presence of LTL, the broadening is clearly identified in their ^31^P NMR spectra giving a definitive proof of their preservation. This result reveals the more important role for LTL than that suggested by the FTIR experiments.

**Figure 5:**
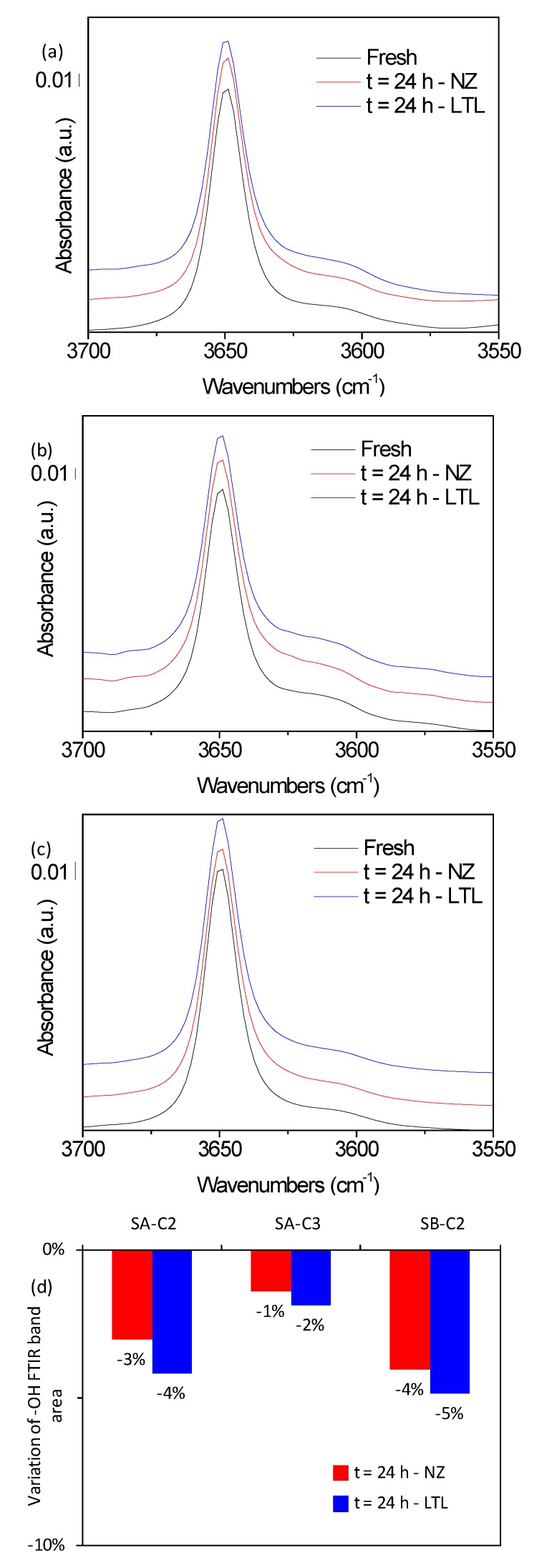
FTIR band vibrations of (**a**) SA-C2, (**b**) SA-C3, (**c**) SB-C2, in their fresh form (black) or following 24 h of oxidation at 150 °C in absence (red) or presence (blue) of LTL nanozeolite. (**d**) Evolution of –OH FTIR band area following oxidation.

In the absence of zeolite, the ZDDP is decomposed and the bands recorded by FTIR correspond to the decomposition products possessing P–O–C bonds, while in the presence of LTL nanocrystals, the ZDDP structure is preserved with limited dissociation being detected. Besides, the ZDDP additive present in lubricants SB-C2 and SB-C3 was completely decomposed with no influence of zeolite being detected, which is in agreement with the results obtained by FTIR. A different behavior was recorded for the phenol antioxidant that displayed a high thermal stability both in the presence and in the absence of zeolite. A minor loss, below 5%, was recorded for the –OH FTIR band for samples SA-C2, SA-C3, and SB-C2 (Figure 5), meanwhile, no modification in the ^1^H NMR profiles was identified (Figure S7).

**Figure 6:**
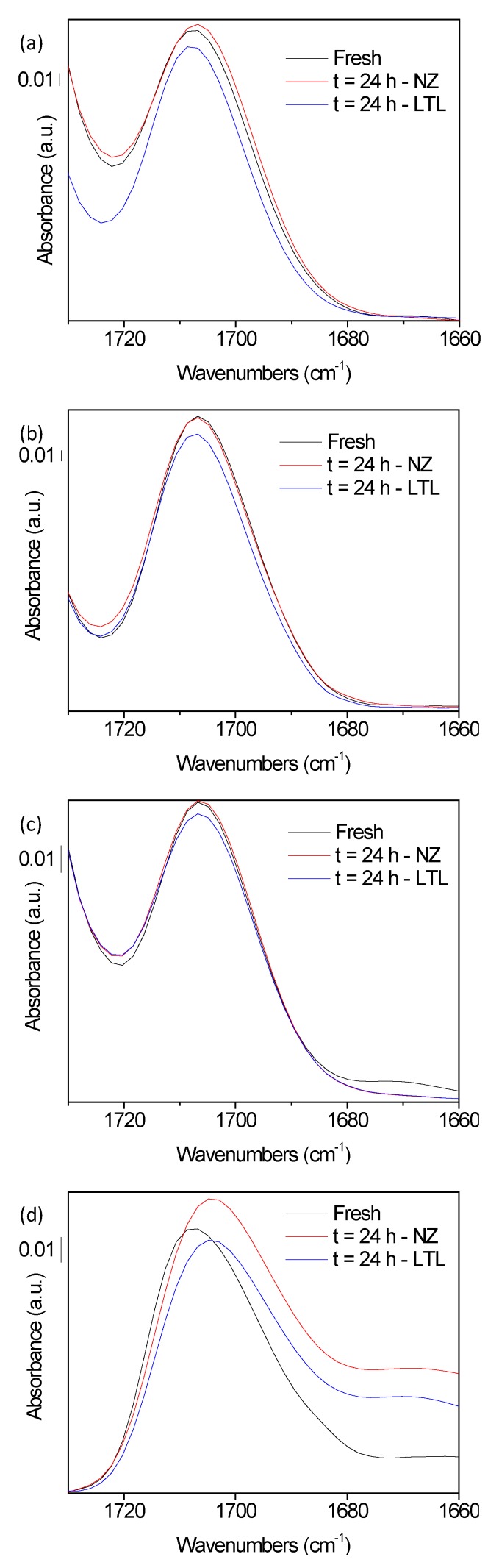
C=O FTIR band vibrations at 1705 cm^−1^ of (**a**) SA-C2, (**b**) SA-C3, (**c**) SB-C2, and (**d**) SB-C3 in their fresh form (black) or following 24 h of oxidation at 150 °C in absence (red) or presence (blue) of LTL nanozeolite.

**Figure 7:**
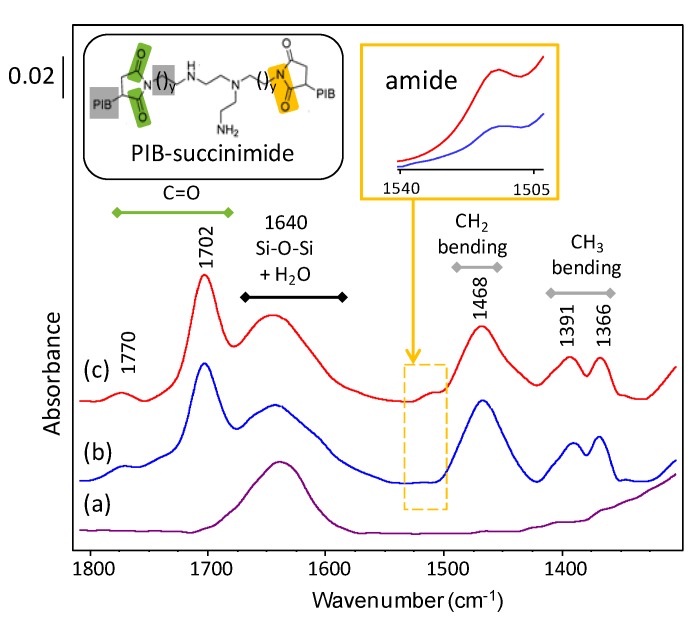
FTIR spectra of (**a**) fresh LTL and LTL extracted after 24 h of heating at 150 °C from (**b**) SA-C3, and (**c**) SA-C2 lubricant samples. Inset, one possible structure of PIB-succinimide.

In contrast to the highly stable fully synthetic base oils studied, the more fragile performance additive can undergo oxidation leading to the formation of aldehydes, ketones and carboxylic acids. They are favoring the progressive oxidation of the performance additives, and lead to formation of heavy polymers (soot) which provokes several problems such as increase of lubricant viscosity and blocking of oil filters. The FTIR band at 1705 cm^−1^ corresponds to the C=O vibrations of the oxidation products (aldehydes, ketones, and carboxylic acid) is found to slightly broaden for the lubricants aged in the absence of zeolite nanocrystals (Figure 6). A deconvelution of this region showed an increase of 6–8% (Figures S8 and S9) which is corresponding to the secondary oxidation products. In the presence of zeolite nanocrystals, lower values were recorded due to the slower formation of oxidation products and to their subsequent adsorption on the zeolite scavengers. The negative values recorded for samples SA-C2 and SA-C3 can be attributed to the partial adsorption of PIB-succinimide that presents C=O vibrations in the same region. Indeed, the FTIR spectra of the LTL zeolite samples separated from the lubricants revealed the presence of PIB-succinimide vibrations vibrations [23,32] (Figure 7 and Figure S10). The ratio (1705/1770) between the two bands at 1705 cm^−1^ and 1770 cm^−1^ corresponding to this additive is found to be higher in the zeolite powder samples extracted from oxidized lubricants than in the fresh lubricants, suggesting the absorption of additional “C=O” containing species from oxidation products on the zeolite. Noteably, the zeolite FTIR structural bands located between 400 and 1000 cm^−1^ were all intact after their use in the oxidation experiments, which reflects the high stability of the zeolite structure under the conditions used (Figure S10). This was further highlighted by the XRD patterns showing identical features for zeolite samples recorded before and after being involved in the oil oxidation process (Figure S1).

## 4. Conclusions

LTL nanozeolite prepared from organic free precursor suspensions with no post-synthetic treatment is used as a proactive agent to extend lubricant lifetime and thus reduce the environmental and economic impact from their disposal and recycling. The zeolite demonstrated a dual action: (1) it showed a high capability in preventing the depletion of ZDDP (zinc dialkyldithiophosphate) additive at elevated temperatures as proven by ^31^P NMR and FTIR spectroscopy, and (2) it acted as a scavenger that collects the oxidation products from the lubricants to prevent the further depletion of additives and oxidation of base oil leading to the formation of heavy molecules (soot particles).

## Supplementary Materials

Available online at https://www.mdpi.com/1996-1944/12/17/2830/s1, Figure S1: XRD patterns of fresh LTL nanosized zeolite (red) and LTL nanosized zeolite separated from lubricant oxidized at 150 °C for 24 h (black), Figure S2: Particle size distribution of LTL nanosized zeolite crystals determined by dynamic light scattering (DLS), Figure S3: N_2_ adsorption (full circles) and desorption (empty circles) isotherm of LTL nanosized zeolite crystals, Figure S4: (up) ^1^H NMR spectra of the fresh lubricants; (down) zoom on the aromatic region of the spectra, Figure S5: FTIR spectra of the fresh lubricants, Figure S6: Evolution of the FTIR band area of P–O–C bonds for lubricants oxidized at 150 °C for 16 h and 24 h in the presence (LTL) or absence (NZ) of LTL nanosized zeolite. *Inset*: FTIR spectra of SA-C3 before oxidation (black) and after 24 h of oxidation in the presence (blue) and absence (red) of LTL nanosized zeolite, Figure S7: ^1^H NMR spectra of SA-C2, before (blue) and after 24 h of oxidation at 150 °C in the absence (green) or presence (violet) of LTL nanosized zeolite, Figure S8: Deconvolution of C=O FTIR band vibrations of the lubricant samples (a) SA-C2 Fresh, (b) SA-C2, no zeolite, 150 °C, 24 h, (c) SA-C2, LTL, 150 °C, 24 h, (d) SA-C3 Fresh, (e) SA-C3, no zeolite, 150 °C, 24 h, (f) SA-C3, LTL, 150 °C, 24 h, (g) SB-C2 Fresh, (h) SB-C2, no zeolite, 150 °C, 24 h, (i) SB-C2, LTL, 150 °C, 24 h, (j) SB-C3 Fresh, (k) SB-C3, no zeolite, 150 °C, 24 h, and (l) SB-C3, LTL, 150 °C, 24 h, Figure S9: Evolution of the FTIR band area of C=O bonds at 1705 cm^−1^ for lubricants oxidized at 150 °C for 24 h in the presence (blue) and absence (red) of LTL nanosized zeolite, Figure S10: FTIR spectra of (a) fresh LTL and LTL zeolite extracted after 24 h of treatment at 150 °C from (b) SA-C3, and (c) SA-C2 lubricant samples, Table S1: Viscosity of lubricants used in the study measured at 25 °C.
