# Supplementary material for: Zeolite Nanocrystals Protect the Performance of Organic Additives and Adsorb Acid Compounds during Lubricants Oxidation
Source: Materials (Basel). 2019 Sep 3;12(17):2830. doi: 10.3390/ma12172830 (PMC6747958; doi:10.3390/ma12172830)
Supplement: Supplementary file 1 [file materials-12-02830-s001.pdf]

materials

Supplementary information

Article

Zeolite Nanocrystals Protect the Performance of Organic Additives and Adsorb Acid Compounds during Lubricants Oxidation

Moussa Zaarour [1,*], Hussein El Siblani [1], Nicolas Arnault [2], Philippe Boullay [3] and Svetlana Mintova [1,*]

[1] Normandie Univ, ENSICAEN, UNICAEN, CNRS, Laboratoire Catalyse et Spectrochimie, 14050 Caen, France; elsiblani@ensicaen.fr

[2] Sogefi Group, Parc Ariane IV, 7 Avenue du 8 mai 1945, 78286 Guyancourt CEDEX, France; nicolas.arnault@sogefigroup.com

[3] Normandie Univ, ENSICAEN, UNICAEN, CNRS, CRISMAT, 14000 Caen, France; philippe.boullay@ensicaen.fr

* Correspondence: moussa.zaarour@ensicaen.fr (M.Z.), mintova@ensicaen.fr (S.M.)

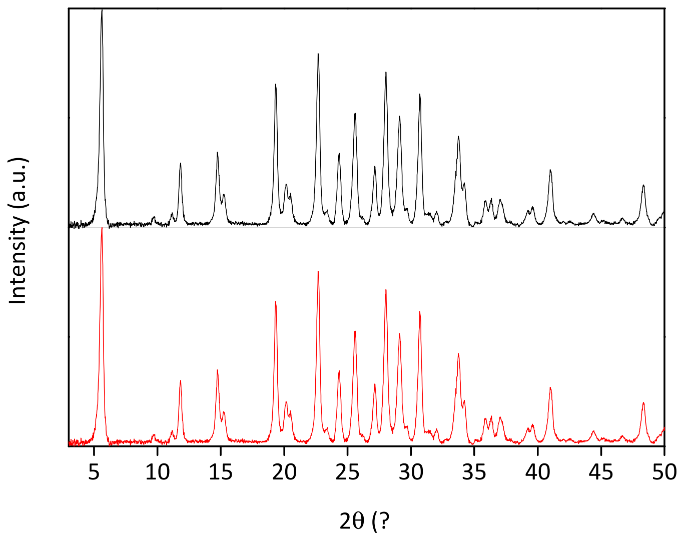

Figure S1. XRD patterns of fresh LTL nanosized zeolite (red) and LTL nanosized zeolite separated from lubricant oxidized at 150 °C for 24 h (black).

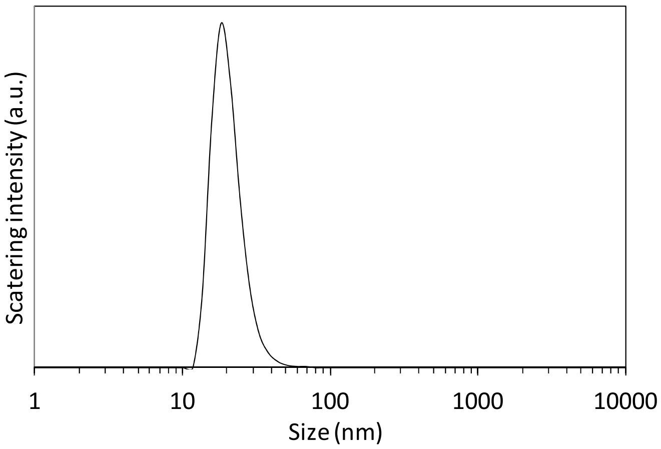

Figure S2. Particle size distribution of LTL nanosized zeolite crystals determined by dynamic light scattering (DLS).

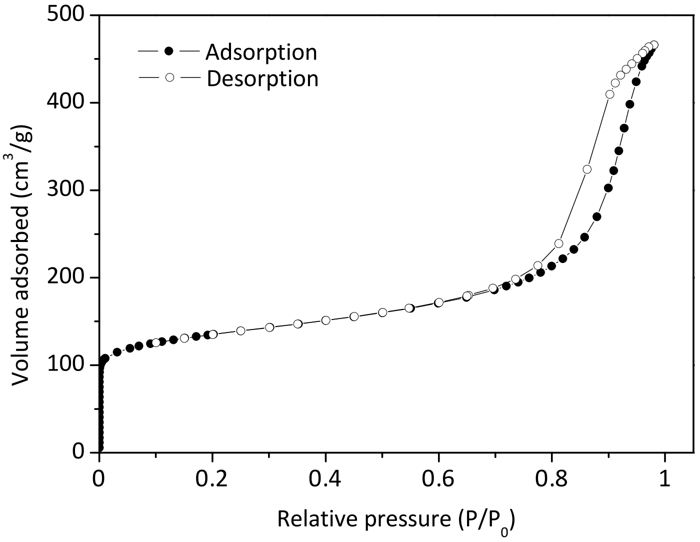

Figure S3. N_2 adsorption (full circles) and desorption (empty circles) isotherm of LTL nanosized zeolite crystals.

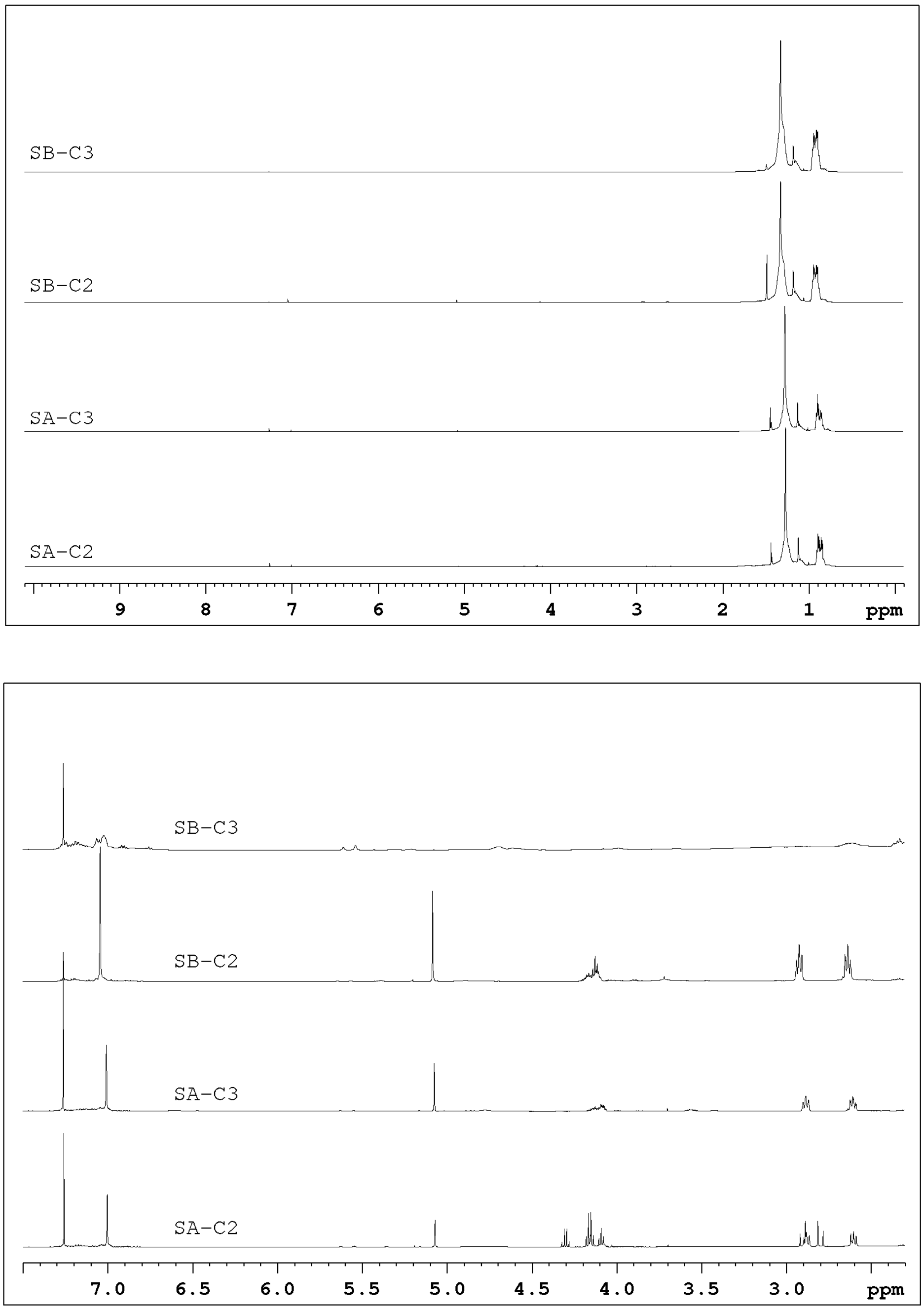

Figure S4. (up) ^{1}H NMR spectra of the fresh lubricants; (down) zoom on the aromatic region of the spectra.

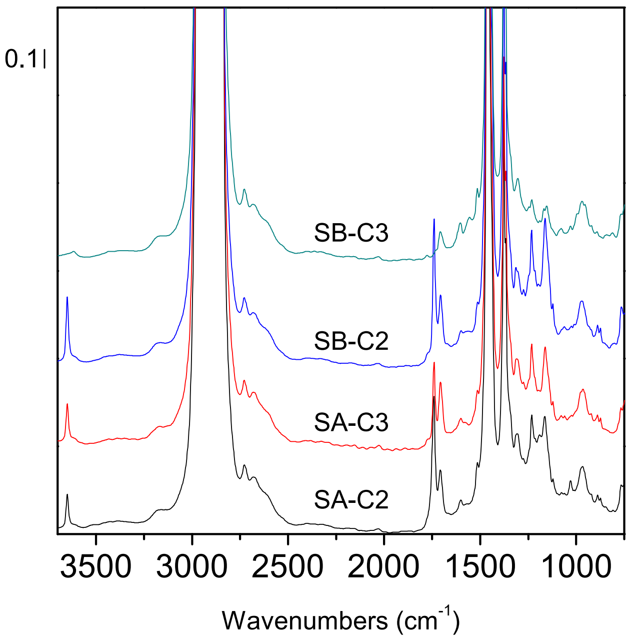

Figure S5. FTIR spectra of the fresh lubricants.

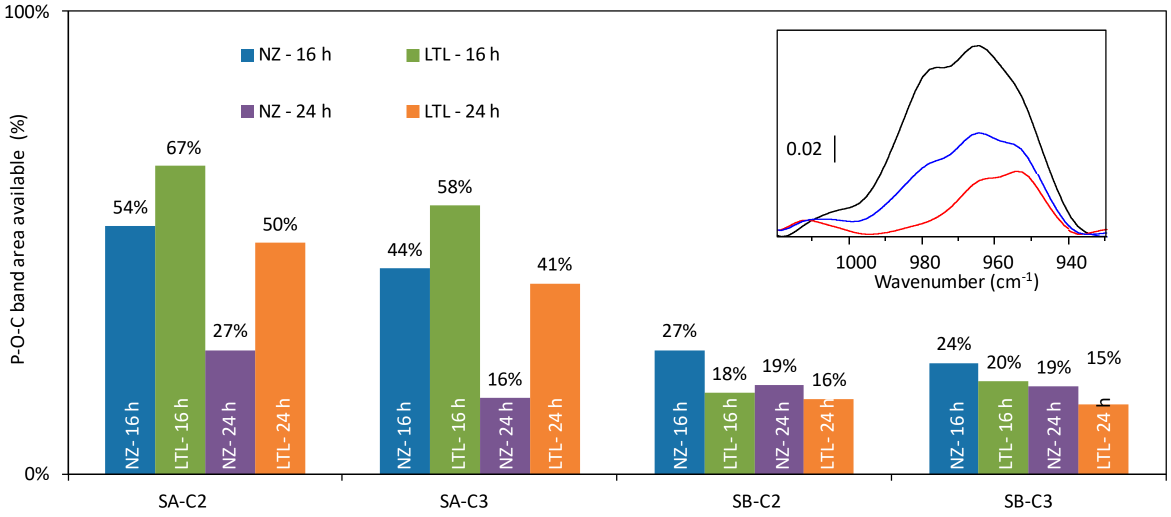

Figure S6. Evolution of the FTIR band area of P-O-C bonds for lubricants oxidized at 150 °C for 16 h and 24 h in the presence (LTL) or absence (NZ) of LTL nanosized zeolite. *Inset*: FTIR spectra of SA-C3 before oxidation (black) and after 24 h of oxidation in the presence (blue) and absence (red) of LTL nanosized zeolite.

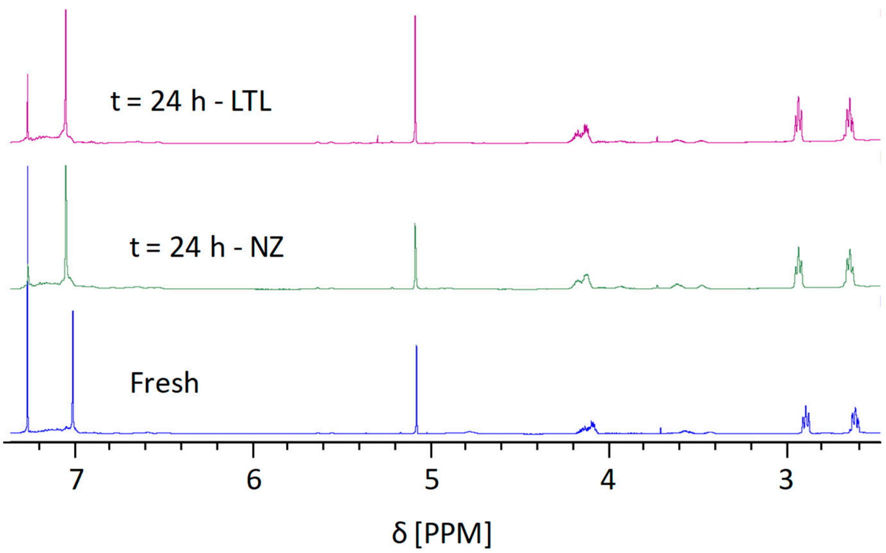

Figure S7. ^{1}H NMR spectra of SA-C2, before (blue) and after 24 h of oxidation at 150 °C in the absence (green) or presence (violet) of LTL nanosized zeolite.

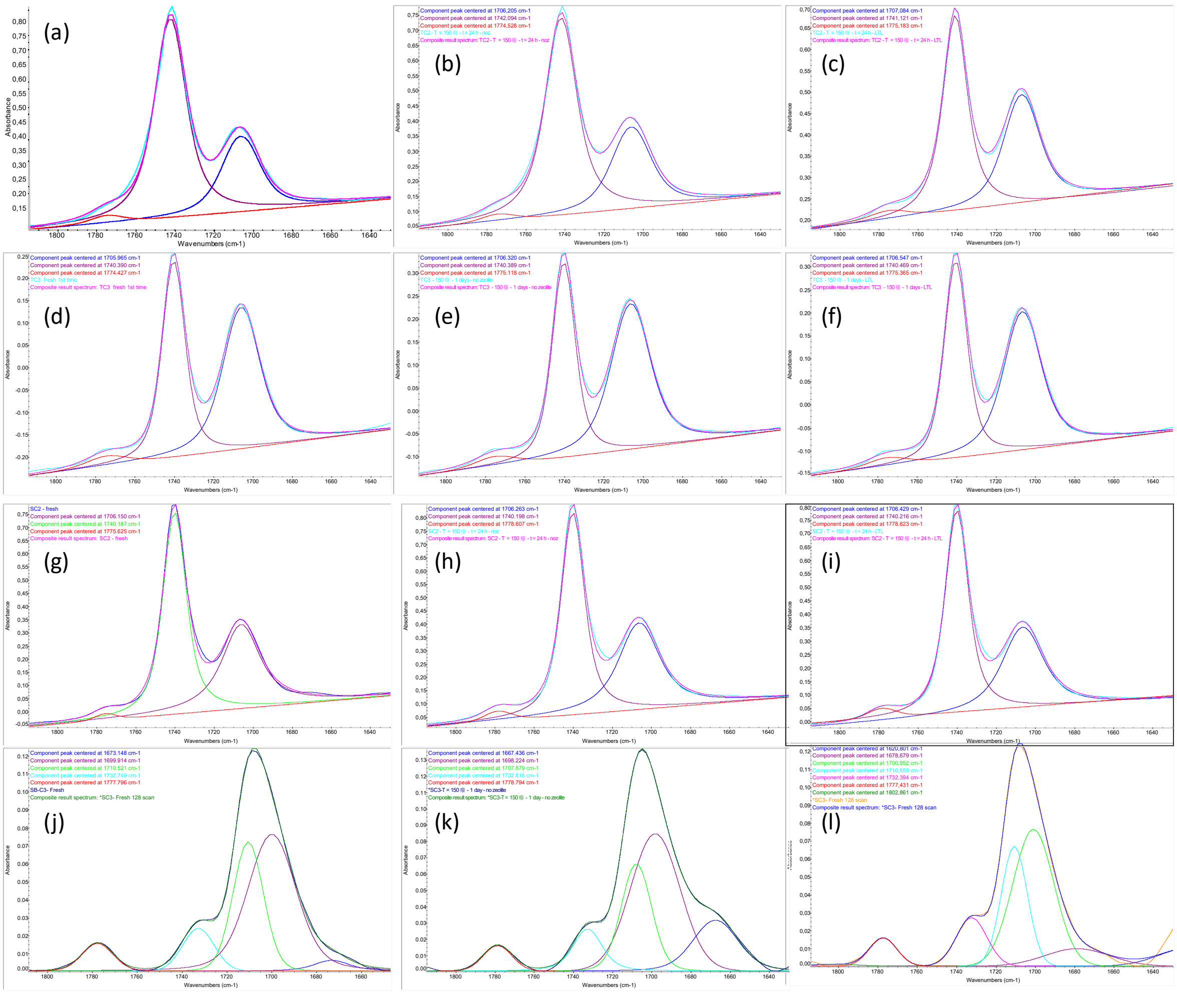

Figure S8. Deconvolution of C=O FTIR band vibrations of the lubricant samples (a) SA-C2 Fresh, (b) SA-C2, no zeolite, 150 °C, 24 h, (c) SA-C2, LTL, 150 °C, 24 h, (d) SA-C3 Fresh, (e) SA-C3, no zeolite, 150 °C, 24 h, (f) SA-C3, LTL, 150 °C, 24 h, (g) SB-C2 Fresh, (h) SB-C2, no zeolite, 150 °C, 24 h, (i) SB-C2, LTL, 150 °C, 24 h, (j) SB-C3 Fresh, (k) SB-C3, no zeolite, 150 °C, 24 h, and (l) SB-C3, LTL, 150 °C, 24 h.

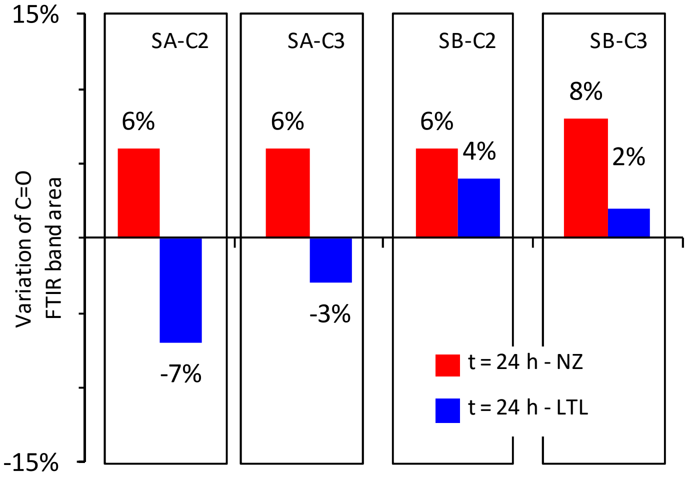

Figure S9. Evolution of the FTIR band area of C=O bonds at 1705 cm^{-1} for lubricants oxidized at 150 °C for 24 h in the presence (blue) and absence (red) of LTL nanosized zeolite.

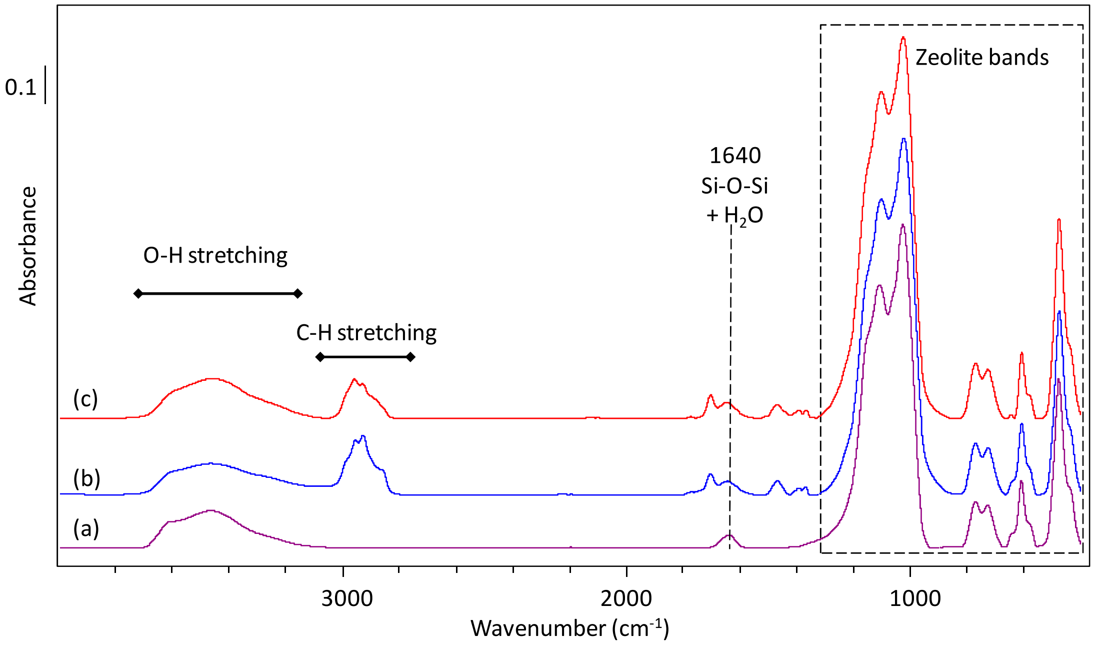

Figure S10. FTIR spectra of (a) fresh LTL and LTL zeolite extracted after 24 h of treatment at 150 °C from (b) SA-C3, and (c) SA-C2 lubricant samples.

Table S1. Viscosity of lubricants used in the study measured at 25 °C.

Reference	Supplier	Grade	Viscosity η (Pa·s)
SA-C2	SA	C2	0.1032 ± 0.00063
SA-C3	SA	C2	0.116655 ± 0.001455
SB-C2	SB	C3	0.10197 ± 0.00196
SB-C3	SB	C3	0.10519 ± 0.00085
